# Antimicrobial activities of commercial essential oils and their components against food-borne pathogens and food spoilage bacteria

**DOI:** 10.1002/fsn3.116

**Published:** 2014-05-04

**Authors:** Hasika Mith, Rémi Duré, Véronique Delcenserie, Abdesselam Zhiri, Georges Daube, Antoine Clinquart

**Affiliations:** 1 Department of Food Science, Faculty of Veterinary Medicine, FARAH, University of LiègeLiège, Belgium; 2 Department of Food Technology and Chemical Engineering, Institute of Technology of CambodiaPhnom Penh, Cambodia; 3 R&D Department, Pranarom International37 Avenue des Artisans, Ghislenghien, B-7822, Belgium

**Keywords:** Antimicrobial activity, essential oil, food spoilage bacteria, food-borne pathogenic bacteria

## Abstract

This study was undertaken to determine the in vitro antimicrobial activities of 15 commercial essential oils and their main components in order to pre-select candidates for potential application in highly perishable food preservation. The antibacterial effects against food-borne pathogenic bacteria (*Listeria monocytogenes*, *Salmonella* Typhimurium, and enterohemorrhagic *Escherichia coli* O157:H7) and food spoilage bacteria (*Brochothrix thermosphacta* and *Pseudomonas fluorescens*) were tested using paper disk diffusion method, followed by determination of minimum inhibitory (MIC) and bactericidal (MBC) concentrations. Most of the tested essential oils exhibited antimicrobial activity against all tested bacteria, except galangal oil. The essential oils of cinnamon, oregano, and thyme showed strong antimicrobial activities with MIC ≥ 0.125 *μ*L/mL and MBC ≥ 0.25 *μ*L/mL. Among tested bacteria, *P. fluorescens* was the most resistant to selected essential oils with MICs and MBCs of 1 *μ*L/mL. The results suggest that the activity of the essential oils of cinnamon, oregano, thyme, and clove can be attributed to the existence mostly of cinnamaldehyde, carvacrol, thymol, and eugenol, which appear to possess similar activities against all the tested bacteria. These materials could be served as an important natural alternative to prevent bacterial growth in food products.

## Introduction

Pathogenic and food spoilage bacteria have been considered as the primary causes of food-borne diseases and food quality deterioration in both developed and developing countries. In order to assure the food safety and to extend the shelf life of food products, additions of chemical preservative agents into food products or decontamination treatments via physical, chemical or biological process or their combinations have been widely applied in food industries (Brul and Coote 1999; Gould 2000). However, critical concerns have been raised due to limitations of treatment processes and since survival of environment-adapted bacteria after treatment processes may lead to high resistance of bacteria such as pathogenic *Escherichia coli* O157:H7, *Listeria monocytogenes*, and some *Salmonella* serovars (Whitney et al. 2007; Hugas and Tsigarida 2008; Rajkovic et al. 2009). The different diseases such as campylobacteriosis, listeriosis, hemorrhagic colitis, and salmonellosis caused by food-related pathogenic bacteria were still reported (Newell et al. 2010; EFSA and ECDC 2011). In highly perishable foods such as meat and meat products, spoilage bacteria contribute to shorten the shelf life by causing off-odors, off-flavors, discoloration, gas production, and slime production (Ercolini et al. 2009). Need for natural alternative is due to consumers' preference for fewer chemicals and more natural foods. Regulatory approval is easy (GRAS) for being natural antimicrobials. Apparently, essential oils have been considered as potential alternatives. These secondary metabolites can be obtained from flowers, buds, seeds, leaves, bark, herbs, fruits, and roots of plants through expression, solvent extraction, steam or hydro distillation. These volatile oils containing bioactive compounds were known for biological activity, remarkably antioxidant activity (Mechergui et al. 2010; Viuda-Martos et al. 2010) and antimicrobial activity against food-borne pathogens and food spoilage bacteria (Burt 2004; Dadalioğlu and Evrendilek 2004; Oussalah et al. 2007; Sarac and Ugur 2008; Viuda-Martos et al. 2008; Ruiz-Navajas et al. 2012). Several studies on application of essential oils as antimicrobials have been conducted and shown to increase the safety and shelf life of food products besides being used as flavoring agent in foods (Burt 2004; Bajpai et al. 2012).

In the study of Oussalah et al. (2007), some commercial oils from Canadian supplier such as *Corydothymus capitatus*, *Cinnamomum cassia*, *Cinnamomum verum*, and *Origanum heracleoticum* were observed to strongly inhibit pathogenic *Staphylococcus aureus*, *L. monocytogenes*, *E. coli* O157:H7, and *Salmonella* Typhimurium on Brain Heart Infusion (BHI) agar. Out of 21 essential oils from an Indian producer, *Cinnamomum zeylanicum*, *Eugenia caryophyllata*, and *Citrus aurantium* oils were the most effective in inhibiting some tested bacterial strains of *Bacillus subtilis*, *S. aureus*, *E. coli*, *Klebsiella pneumoniae*, *Pseudomonas aeruginosa*, and *Pseudomonas vulgaris* on Mueller-Hinton (MHA) agar (Prabuseenivasan et al. 2006). *Syzygium aromaticum* oil was the strongest among the 10 commercial oils tested in inhibiting the growth of four strains of *E. coli* O157:H7 in BHI broth (Moreira et al. 2005). No antimicrobial activity determination of their main components was included in these works. Some studies claim that oxygenated monoterpenes present in herbs and spices essential oils might also play a major role in their antimicrobial activity. Twenty-one constituents showed variation in antimicrobial activities against 25 bacterial strains including *E. coli*, *Salmonella* Pullorum, *P. aeruginosa*, and *Brochothrix thermosphacta* when assayed by agar well diffusion method using Iso-Sensitest agar (Dorman and Deans 2000). Carvacrol, cinnamic acid, eugenol, and thymol were also tested against *E. coli* and *S*. Typhimurium using Bioscreen for MIC determination in Luria broth (LB) (Olasupo et al. 2003). Kotan et al. (2007) reported that only linalool, nerol, terpinen-4-ol, *α*-terpineol, and fenchol, out of 21 oxygenated monoterpenes were mostly active against 63 bacterial strains including *Salmonella* Enteritidis, *S*. Typhimurium and *P. aeruginosa* on nutrient agar (NA) using paper disk diffusion method. However, the outputs reported from these different studies are difficult to compare directly by different test methods, diverse bacterial strains, culture media, and antimicrobial sample sources.

Nowadays the essential oils or extracts from daily-used-culinary herbs and spices are commercially acquired with Europe Union as the world's biggest importer (UNIDO and FAO 2005). In spite of all the information available on several essential oils, the investigation dealing with this kind of commercial products, which are generally the ones used by mostly flavor and fragrance industries, especially food and beverage industries, have been inadequate. Therefore, the aim of this study was to determine the in vitro antimicrobial activity of commercial essential oils and standard constituents against food-borne pathogens and food spoilage bacteria in order to preselect potential candidates for application in food preservation.

## Materials and Methods

### Essential oils

In this study, the essential oils provided by Pranarom International (Ghislenghien, Belgium) and Lionel Hitchen Limited (Hampshire, United Kingdom) were screened for antimicrobial activity. The list of essential oils and their properties are given in Table 1. Some individual constituents (carvacrol, *trans*-cinnamaldehyde, eugenol, linalool, and thymol) commonly found in these essential oils were purchased from Sigma-Aldrich Chemie GmbH (Steinheim, Germany). These oils were stored at 4°C before use.

**Table 1:** List of essential oils and their properties

Botanical species	Common name	Family	Part	Main composition (%)^1^	Manufacturers
*Cinnamomum cassia*	Chinese cinnamon	Lauraceae	Leaf-branch	E-cinnamaldehyde (77.90), *trans*-o-methoxy-cinnamaldehyde (10.50)	Pranarom
*Cinnamomum verum*	Ceylon cinnamon	Lauraceae	Bark	E-cinnamaldehyde (63.56), cinnamyl acetate (8.33)	Pranarom
*Coriandrum sativum*	Coriander	Apiaceae	Fruit	Linalool (70.07), camphor (5.52), *α*-pinene (4.86)	Pranarom
*Cymbopogon flexuosus*	Indian lemongrass	Gramineae	Herb grass	NA	Lionel Hitchen
*Cymbopogon nardus*	Ceylon citronella	Gramineae	Herb grass	Geraniol (24.08), camphene (9.01), geranyl acetate (8.81)	Pranarom
*Eugenia caryophyllus*	Clove	Myrtaceae	Bud	Eugenol (84.75), eugenyl acetate (7.12), *β*-caryophyllene (4.60)	Pranarom
*Kaempferia galanga*	Aromatic ginger	Zingiberaceae	Rhizome	NA	Lionel Hitchen
*Origanum compactum*	Oregano	Lamiaceae	Flowering plant	Carvacrol (46.37), thymol (13.70), p-cymene (13.33)	Pranarom
*Origanum heracleoticum*	Greek oregano	Lamiaceae	Flowering plant	Carvacrol (68.14), thymol (7.47), *γ*-terpinene (6.06)	Pranarom
*Origanum majorana*	Sweet marjoram	Lamiaceae	Flowering plant	Terpinene-4-ol (24.21), *α*-terpinene (8.44), sabinene (7.12)	Pranarom
*Salvia officinalis*	Dalmatian sage	Lamiaceae	Flowering plant	NA	Lionel Hitchen
*Salvia sclarea*	Clary sage	Lamiaceae	Flowering plant	Linalyl acetate (62.38), linalool (21.47), *α*-terpineol (2.45)	Pranarom
*Thymus capitatus*	Oregano	Lamiaceae	Flowering plant	NA	Lionel Hitchen
*Thymus mastichina*	Spanish marjoram	Lamiaceae	Flowering plant	NA	Lionel Hitchen
*Thymus vulgaris thymoliferum*	Common thymol thyme	Lamiaceae	Flowering plant	Thymol (39.74), p-cymene (18.74), *γ*-terpinene (11.12)	Pranarom

NA, not available.

1 Based on the data of the gas-chromatography analysis of essential oils provided by manufacturers.

### Bacterial strains

To assess the antibacterial properties of the test samples, six strains of pathogenic bacteria were used in the study: *L. monocytogenes* NCTC 11994, *L. monocytogenes* S0580 (isolated from raw pork meat), *S*. Typhimurium ATCC 14028, *S*. Typhimurium S0584 (isolated from pig carcass), *Escherichia coli* O157:H7 ATCC 35150 and *E. coli* O157:H7 S0575 (isolated from minced beef). The S0580, S0584, and S0575 strains have been isolated by internal laboratory for microbiological analysis. Other two strains of spoilage bacteria *B. thermosphacta* ATCC 11509 and *Pseudomonas fluorescens* ATCC 13525 were also included. Bacterial strains were grown in BHI broth and incubated at 37°C for 24 h, except for *P. fluorescens* ATCC 13525 (30°C) and *B. thermosphacta* ATCC 11509 (22°C).

### Antibacterial activity assays

As a preliminary step, the antibacterial activities of the essential oils were determined by using paper disk diffusion method to screen the efficacy of essential oils among all samples. The essential oils were diluted with analytical grade ethanol at the following concentration 1, 1/1, 1/10, 1/20, and 1/40 (v/v). A volume of 20 *μ*L of each concentration was, respectively, impregnated into the paper disk with 6 mm diameter (Biomérieux, Marcy-l'Etoile, France), and then placed onto Mueller-Hinton agar (MHA) plates (Oxoid, Badhoevedorp, Netherlands), which were previously inoculated on the surface agar with 200 *μ*L of 10^6^ cfu/mL suspension for each tested bacterium. Ethanol was used as a control. Some individual components (carvacrol, cinnamaldehyde, eugenol, linalool, and thymol), frequently present as major component in essential oils, were also tested. Three standard reference antibiotics, ampicillin (10 *μ*g/disk), chloramphenicol (30 *μ*g/disk), and streptomycin (10 *μ*g/disk), were used as reference controls for the tested bacteria. The plates were then incubated at 37°C for 24 h for *L. monocytogenes*, *S*. Typhimurium and *E. coli* O157:H7, and at 30°C for 24 h for *P. fluorescens*, and at 22°C for 48 h for *B. thermosphacta*. The antibacterial activity was evaluated by measuring the diameter of inhibitory zones in millimeters using digital calliper Top Craft (Globaltronics GmbH & Co. KG, Hamburg, Germany) and the means were expressed as the results of five determinations.

### Determination of minimum inhibitory and bactericidal concentrations

The essential oils, which exhibited the best antimicrobial activity in the paper disk diffusion assay, and some individual constituents, were selected for determining the minimum inhibitory concentration (MIC) and the minimum bactericidal concentration (MBC) using broth dilution method. One colony of each bacterial strain was sampled with a loop, then inoculated in 25 mL BHI broth and incubated for 18–24 h at 37°C in order to get a bacterial suspension of 10^9^ cfu/mL. Only *P. fluorescens* and *B. thermosphacta* were incubated at, respectively, 30 and 22°C. Each stock solution was diluted with buffered peptone water (Oxoid) to obtain 10^5^ cfu/mL bacterial suspensions. Serial dilutions of essential oils (0.125–5 *μ*L/mL) were prepared with BHI broth medium in test tube and mixed with bacterial suspensions to give a volume of 4 mL and a final concentration of bacteria of approximately 5 × 10^4^ cfu/mL. Final solutions were incubated at the temperature mentioned earlier. The MIC was considered as the lowest concentration that prevented the visible growth. The MBC was determined by subculturing 100 *μ*L from each negative test tube onto plate count agar (PCA) plates. MBC was defined as the lowest concentration resulting in a negative subculture or giving presence of only one colony after incubation. The experiments were carried out in four replicates.

### Statistical analysis

The mean values ± standard deviations were calculated. Analysis of variance was performed on the basis of mean values to determine the significant difference between essential oils at *P* ≤ 0.05. Statistical analysis was undertaken using the SAS version 9.1 (Cary, NC).

## Results and Discussion

### Antimicrobial activity of essential oils

The antibacterial activities of essential oils against eight bacterial strains are summarized in Tables 2–5. The results represent the diameter of inhibition zone including diameter of paper disk (6 mm). A broad variation in antimicrobial properties of the analyzed oils was observed in the study. The essential oils of *C. cassia*, *C. verum*, *Origanum compactum*, *O. heracleoticum*, *Thymus capitatus*, and *Thymus vulgaris thymoliferum* showed consistently strong antimicrobial activity against tested bacteria at different diluted concentrations (1, 1/2, 1/10, 1/20, and 1/40), whereas *Cymbopogon flexuosus* essential oil showed only strong activity against Gram-positive bacteria. Essential oil of *Eugenia caryophyllus* showed consistently moderate activity against all tested bacteria. On the other hand, *Cymbopogon nardus* and *Salvia sclarea* oils were weak or failed to inhibit the growth of Gram-negative bacteria, while *Kaempferia galanga* oil showed no antimicrobial activity against any of the tested bacterial strains. Interestingly, oil of *Origanum majorana* was more active against Gram-negative bacteria than Gram-positive bacteria. Overall, *L. monocytogenes* NCTC 11994, *L. monocytogenes* S0580, and *B. thermosphacta* ATCC 11509 were inhibited by 14 oils, followed by *S*. Typhimurium S0584 (13 oils), *S*. Typhimurium ATCC 14028 and *E. coli* O157:H7 S0575 (12 oils), *E. coli* O157:H7 ATCC 35150 (11 oils) and *P. fluorescens* ATCC 13525 (10 oils). Obviously, *P. fluorescens* showed least susceptibility to the tested essential oils. Generally, the Gram-positive bacteria were more sensitive to essential oils or antibacterial compounds than Gram-negative bacteria, which is in a good agreement with previous reports (Russell 1995; Smith-Palmer et al. 1998; Dorman and Deans 2000; Burt 2004; Shan et al. 2007). This resistance could be ascribed to the structure of the cellular walls of Gram-negative bacteria, mainly with regard to the presence of lipoproteins and lipopolysaccharides that form a barrier to restrict entry of hydrophobic compounds (Russell 1995; Cox and Markham 2007).

**Table 2:** Antimicrobial activity of essential oils against *Listeria monocytogenes* NCTC 11994 and *L. monocytogenes* S0580 by paper disk diffusion method

	*L. monocytogenes* NCTC 11994					*L. monocytogenes* S0580				
		
	1^1^	1/2^1^	1/10^1^	1/20^1^	1/40^1^	1	1/2	1/10	1/20	1/40
Essential oils	Diameter of inhibition zone (mm)^2^									
*Cinnamomum cassia*	33.8 ± 0.2^a^	30.1 ± 0.5^a^	25.4 ± 0.1^a^	14.6 ± 0.8^a^	8.9 ± 0.3^a^	34.1 ± 0.3^a^	28.4 ± 0.8^a^	23.1 ± 0.4^a^	16.6 ± 0.7^a^	8.5 ± 0.4^a^
*Cymbopogon flexuosus*	34.8 ± 0.3^b^	24.3 ± 0.8^b^	9.1 ± 0.1^b^	8.2 ± 0.2^b^	7.1 ± 0.1^b^	34.0 ± 0.3^a^	26.0 ± 0.4^b^	8.3 ± 0.2^b^	7.9 ± 0.1^b^	–
*Cymbopogon nardus*	11.8 ± 0.6^c^	10.5 ± 0.3^c^	–	–	–	10.8 ± 0.3^b^	8.9 ± 0.3^c^	–	–	–
*Coriandrum sativum*	11.7 ± 0.6^c^	8.0 ± 0.5^d^	–	–	–	10.6 ± 0.4^b^	7.3 ± 0.5^d^	–	–	–
*Cinnamomum verum*	34.0 ± 0.4^a^	28.5 ± 0.2^e^	21.9 ± 0.6^c^	8.7 ± 1.0^c^	–	34.0 ± 0.5^a^	27.9 ± 1.0^e^	17.6 ± 0.8^c^	8.7 ± 0.2^c^	–
*Eugenia caryophyllus*	14.9 ± 0.5^d^	11.3 ± 0.8^f^	8.8 ± 0.5^b^	7.0 ± 0.9^d^	–	14.4 ± 0.1^c^	12.7 ± 0.2^f^	7.0 ± 0.1^d^	–	–
*Kaempferia galanga*	–	–	–	–	–	–	–	–	–	–
*Origanum compactum*	26.8 ± 0.6^e^	22.6 ± 0.3 ^g^	11.1 ± 0.3^d^	8.7 ± 0.1^c^	7.5 ± 0.3^b^	26.8 ± 0.5^d^	24.3 ± 0.7 ^g^	11.4 ± 0.4^e^	8.7 ± 0.2^c^	7.8 ± 0.1^b^
*Origanum heracleoticum*	31.5 ± 0.3^f^	25.9 ± 0.3 ^h^	18.1 ± 0.2^e^	12.7 ± 0.2^e^	10.0 ± 0.2^c^	31.1 ± 0.5^e^	27.4 ± 0.5^e^	17.6 ± 0.3^c^	12.8 ± 0.1^d^	9.9 ± 0.1^c^
*Origanum majorana*	10.9 ± 0.2 ^g^	–	–	–	–	11.0 ± 0.2^b^	7.0 ± 0.1^d^	–	–	–
*Salvia officinalis*	10.3 ± 0.2 ^g^	8.4 ± 0.4^d^	–	–	–	8.8 ± 0.2^f^	7.2 ± 0.3^d^	–	–	–
*Salvia sclarea*	10.4 ± 0.9 ^g^	7.2 ± 0.3^i^	–	–	–	9.8 ± 0.3 ^g^	7.0 ± 0.1^d^	–	–	–
*Thymus capitatus*	30.2 ± 0.7 ^h^	23.6 ± 0.3^j^	15.6 ± 0.3^f^	11.8 ± 0.2^f^	9.9 ± 0.2^c^	29.3 ± 0.8 ^h^	24.6 ± 0.6 ^g^	16.9 ± 0.4^f^	12.5 ± 0.2^d^	10.2 ± 0.2^c^
*Thymus mastichina*	9.5 ± 0.4^i^	–	–	–	–	10.8 ± 1.1^b^	7.0 ± 0.1^d^	–	–	–
*Thymus vulgaris thymoliferum*	30.3 ± 0.6 ^h^	27.5 ± 0.7^k^	13.0 ± 0.4 ^g^	9.8 ± 0.4 ^g^	8.0 ± 0.1^d^	32.6 ± 0.4^i^	29.0 ± 0.4 ^h^	13.6 ± 0.5 ^g^	9.9 ± 0.2^e^	8.3 ± 0.1^a^
Ampicillin^3^	33.7 ± 1.2	ND	ND	ND	ND	33.4 ± 0.4	ND	ND	ND	ND
Chloramphenicol^3^	27.8 ± 1.0	ND	ND	ND	ND	26.7 ± 0.6	ND	ND	ND	ND
Streptomycin^3^	18.9 ± 0.6	ND	ND	ND	ND	21.6 ± 0.4	ND	ND	ND	ND
Blank control (ethanol)	–	–	–	–	–	–	–	–	–	–

(–) Diameter of inhibitory zone <7 mm considered as no antimicrobial activity. ND, not determined.

1 Concentrations (1, 1/2, 1/10, 1/20, 1/40) used were v/v.

2 Values are mean diameter of inhibitory zone (mm) ±SD of five replicates, followed by different letters in column are significantly different (*P* < 0.05). The diameter of paper disk (6 mm) is included.

3 Ampicillin (10 *μ*g), chloramphenicol (30 *μ*g), and streptomycin (10 *μ*g) used as positive control.

**Table 3:** Antimicrobial activity of essential oils against *Salmonella* Typhimurium ATCC 14028 and *S*. Typhimurium S0584 using paper disk diffusion method

	*S*. Typhimurium ATCC 14028					*S*. Typhimurium S0584				
		
	1^1^	1/2^1^	1/10^1^	1/20^1^	1/40^1^	1	1/2	1/10	1/20	1/40
Essential oils	Diameter of inhibition zone (mm)^2^									
*Cinnamomum cassia*	27.3 ± 0.5^a^	23.0 ± 0.7^a^	18.6 ± 0.6^a^	12.0 ± 0.6^a^	8.8 ± 0.3^a^	28.2 ± 1.2^a^	22.2 ± 0.8^a^	19.3 ± 0.8^a^	15.5 ± 0.8^a^	9.1 ± 0.1^a^
*Cymbopogon flexuosus*	9.8 ± 0.2^b^	8.3 ± 0.2^bj^	–	–	–	10.3 ± 0.4^b^	9.4 ± 0.1^b^	–	–	–
*Cymbopogon nardus*	–	–	–	–	–	7.4 ± 0.3^c^	7.0 ± 0.1^c^	–	–	–
*Coriandrum sativum*	10.1 ± 0.8^b^	8.6 ± 0.4^b^	–	–	–	13.9 ± 0.4^d^	12.4 ± 0.3^d^	7.4 ± 0.3^b^	–	–
*Cinnamomum verum*	27.7 ± 0.4^c^	23.7 ± 0.4^c^	16.6 ± 0.4^b^	12.6 ± 0.3^b^	8.1 ± 0.2^b^	28.5 ± 0.7^a^	23.5 ± 0.8^e^	19.8 ± 0.9^af^	13.4 ± 0.2^b^	9.1 ± 0.2^a^
*Eugenia caryophyllus*	15.1 ± 0.2^d^	13.3 ± 0.3^d^	10.8 ± 0.2^c^	7.9 ± 0.1^c^	7.2 ± 0.2^c^	16.7 ± 0.4^e^	14.6 ± 0.4^f^	13.5 ± 0.5^c^	9.1 ± 0.1^c^	7.8 ± 0.1^b^
*Kaempferia galanga*	–	–	–	–	–	–	–	–	–	–
*Origanum compactum*	16.6 ± 0.3^e^	15.1 ± 0.5^e^	11.9 ± 0.6^e^	10.7 ± 0.5^d^	8.9 ± 0.3^a^	23.7 ± 0.5^f^	21.2 ± 0.6^g^	16.6 ± 0.4^d^	11.2 ± 0.2^d^	9.4 ± 0.2^a^
*Origanum heracleoticum*	20.7 ± 0.4^f^	18.2 ± 0.7^f^	15.8 ± 0.4^f^	13.1 ± 0.2^e^	12.3 ± 0.3^d^	27.4 ± 0.9^g^	24.5 ± 0.3^h^	21.7 ± 0.6^e^	19.6 ± 0.5^e^	12.3 ± 0.2^c^
*Origanum majorana*	14.1 ± 0.8^g^	9.7 ± 0.4^g^	7.4 ± 0.1^g^	–	–	23.3 ± 0.8^f^	19.0 ± 1.4^i^	7.6 ± 0.2^b^	7.1 ± 0.1^f^	–
*Salvia officinalis*	7.3 ± 0.3^h^	–	–	–	–	7.3 ± 0.5^c^	–	–	–	–
*Salvia sclarea*	–	–	–	–	–	–	–	–	–	–
*Thymus capitatus*	21.0 ± 1.0^f^	18.8 ± 0.3^h^	14.9 ± 0.2^h^	12.0 ± 0.3^a^	11.1 ± 0.2^e^	26.1 ± 0.3 ^h^	23.8 ± 0.5^e^	20.1 ± 0.5^f^	15.0 ± 0.4^a^	11.9 ± 0.2^c^
*Thymus mastichina*	9.0 ± 0.1^i^	8.1 ± 0.1^i^	–	–	–	9.7 ± 0.6^i^	8.2 ± 0.1^j^	–	–	–
*Thymus vulgaris thymoliferum*	19.3 ± 0.2^j^	16.2 ± 0.3^j^	12.3 ± 0.2^i^	11.0 ± 0.1^d^	9.4 ± 0.2^f^	27.5 ± 0.4 ^g^	23.6 ± 0.5^e^	15.3 ± 0.7^g^	11.4 ± 0.4^d^	10.3 ± 0.4^d^
Ampicillin^3^	28.8 ± 0.3	ND	ND	ND	ND	29.2 ± 0.5	ND	ND	ND	ND
Chloramphenicol^3^	29.0 ± 0.6	ND	ND	ND	ND	28.9 ± 0.8	ND	ND	ND	ND
Streptomycin^3^	16.2 ± 0.4	ND	ND	ND	ND	18.4 ± 0.4	ND	ND	ND	ND
Blank control (ethanol)	–	–	–	–	–	–	–	–	–	–

^(–)^Diameter of inhibitory zone <7 mm considered as no antimicrobial activity. ND, not determined.

1 Concentrations (1, 1/2, 1/10, 1/20, 1/40) used were v/v.

2 Values are mean diameter of inhibitory zone (mm) ±SD of five replicates, followed by different letters in column are significantly different (*P* < 0.05). The diameter of paper disk (6 mm) is included.

3 Ampicillin (10 *μ*g), chloramphenicol (30 *μ*g), and streptomycin (10 *μ*g) used as positive control.

**Table 4:** Antimicrobial activity of essential oils against *Escherichia coli* O157:H7 ATCC 35150 and *E. coli* O157:H7 S0575 using paper disk diffusion method

	*E. coli* O157:H7 ATCC 35150					*E. coli* O157:H7 S0575				
		
	1^1^	1/2^1^	1/10^1^	1/20^1^	1/40^1^	1	1/2	1/10	1/20	1/40
Essential oils	Diameter of inhibition zone (mm)^2^									
*Cinnamomum cassia*	28.1 ± 0.7^a^	18.4 ± 0.6^a^	16.8 ± 0.3^a^	14.2 ± 0.5^a^	9.3 ± 0.5^a^	27.8 ± 0.9^a^	20.2 ± 1.3^a^	19.1 ± 1.2^a^	15.1 ± 0.5^a^	8.5 ± 0.3^a^
*Cymbopogon flexuosus*	9.4 ± 0.1^b^	9.1 ± 0.1^b^	–	–	–	9.8 ± 0.2^b^	7.7 ± 0.1^b^	–	–	–
*Cymbopogon nardus*	–	–	–	–	–	–	–	–	–	–
*Coriandrum sativum*	9.5 ± 0.2^b^	9.1 ± 0.4^b^	–	–	–	11.1 ± 0.3^c^	10.8 ± 0.4^c^	–	–	–
*Cinnamomum verum*	28.1 ± 0.8^a^	24.7 ± 0.2^c^	21.6 ± 0.6^b^	15.6 ± 0.8^b^	8.5 ± 0.4^b^	27.6 ± 0.7^a^	23.5 ± 0.9^d^	21.0 ± 1.1^b^	14.9 ± 0.5^a^	7.9 ± 0.7^b^
*Eugenia caryophyllus*	14.6 ± 0.6^c^	13.1 ± 0.6^d^	11.5 ± 0.2^c^	8.7 ± 0.1^c^	7.5 ± 0.1^c^	14.4 ± 0.4^d^	13.2 ± 0.4^e^	11.5 ± 0.2^c^	9.3 ± 0.4^b^	7.1 ± 0.1^c^
*Kaempferia galanga*	–	–	–	–	–	–	–	–	–	–
*Origanum compactum*	15.4 ± 0.2^d^	14.9 ± 0.5^e^	11.3 ± 0.3^c^	10.1 ± 0.4^d^	8.6 ± 0.0^b^	17.5 ± 0.3^e^	16.3 ± 0.5^f^	12.1 ± 0.8^d^	10.7 ± 0.5^c^	9.2 ± 0.2^d^
*Origanum heracleoticum*	20.2 ± 0.4^e^	19.4 ± 0.5^f^	16.0 ± 0.1^d^	13.7 ± 0.3^e^	11.1 ± 0.2^d^	22.1 ± 0.8^f^	19.7 ± 0.4^g^	16.7 ± 0.6^e^	14.1 ± 0.2^d^	11.2 ± 0.3^e^
*Origanum majorana*	15.6 ± 0.3^d^	14.0 ± 0.5^g^	7.2 ± 0.2^e^	–	–	18.0 ± 0.4^e^	13.9 ± 0.6 ^h^	7.4 ± 0.2^f^	–	–
*Salvia officinalis*	–	–	–	–	–	7.3 ± 0.2^g^	–	–	–	–
*Salvia sclarea*	–	–	–	–	–	–	–	–	–	–
*Thymus capitatus*	20.9 ± 0.7^f^	18.3 ± 0.5^a^	14.5 ± 0.5^f^	12.5 ± 0.3^f^	10.5 ± 0.2^e^	20.7 ± 0.8^h^	18.4 ± 0.4^i^	14.5 ± 0.2^g^	12.6 ± 0.4^e^	10.4 ± 0.1^f^
*Thymus mastichina*	8.5 ± 0.7^g^	7.1 ± 0.1^h^	–	–	–	8.8 ± 0.4^i^	–	–	–	–
*Thymus vulgaris thymoliferum*	17.6 ± 1.0^h^	17.0 ± 0.6^i^	12.3 ± 0.0^g^	11.5 ± 0.2^g^	10.1 ± 0.2^e^	19.2 ± 0.7^j^	17.3 ± 0.5^j^	12.6 ± 0.2^d^	11.4 ± 0.2^f^	10.0 ± 0.3^f^
Ampicillin^3^	20.0 ± 0.1	ND	ND	ND	ND	21.6 ± 0.4	ND	ND	ND	ND
Chloramphenicol^3^	22.3 ± 0.5	ND	ND	ND	ND	22.3 ± 0.7	ND	ND	ND	ND
Streptomycin^3^	23.1 ± 0.3	ND	ND	ND	ND	22.4 ± 0.2	ND	ND	ND	ND
Blank control (ethanol)	–	–	–	–	–	–	–	–	–	–

^(–)^Diameter of inhibitory zone <7 mm considered as no antimicrobial activity. ND, not determined.

1 Concentrations (1, 1/2, 1/10, 1/20, 1/40) used were v/v.

2 Values are mean diameter of inhibitory zone (mm) ±SD of five replicates, followed by different letters in column are significantly different (*P* < 0.05). The diameter of paper disk (6 mm) is included.

3 Ampicillin (10 *μ*g), chloramphenicol (30 *μ*g), and streptomycin (10 *μ*g) used as positive control.

**Table 5:** Antimicrobial activity of essential oils against *Brochothrix thermosphacta* ATCC 11509 and *Pseudomonas fluorescens* ATCC 13525 using paper disk diffusion method

	*B. thermosphacta* ATCC 11509					*P. fluorescens* ATCC 13525				
		
	1^1^	1/2^1^	1/10^1^	1/20^1^	1/40^1^	1	1/2	1/10	1/20	1/40
Essential oils	Diameter of inhibition zone (mm)^2^									
*Cinnamomum cassia*	24.9 ± 0.4^a^	22.7 ± 0.3^a^	19.3 ± 0.2^a^	13.3 ± 0.3^a^	7.3 ± 0.5^a^	23.1 ± 0.6^a^	21.1 ± 1.6^a^	16.1 ± 0.4^a^	11.2 ± 0.7^a^	8.5 ± 0.3^ac^
*Cymbopogon flexuosus*	18.9 ± 0.3^b^	15.8 ± 0.1^b^	9.7 ± 0.4^b^	8.7 ± 0.2^b^	7.7 ± 0.1^a^	9.4 ± 0.0^b^	7.5 ± 0.1^b^	7.2 ± 0.0^b^	–	–
*Cymbopogon nardus*	15.4 ± 0.3^c^	–	–	–	–	–	–	–	–	–
*Coriandrum sativum*	16.3 ± 0.3^d^	13.5 ± 0.5^c^	–	–	–	12.8 ± 0.6^c^	10.5 ± 0.9^c^	7.3 ± 0.2^b^	–	–
*Cinnamomum verum*	24.2 ± 0.7^e^	23.1 ± 0.1^a^	17.2 ± 0.4^c^	12.0 ± 0.3^c^	–	23.6 ± 0.3^d^	21.4 ± 0.3^a^	12.5 ± 0.4^c^	10.4 ± 0.1^b^	8.5 ± 0.2^ac^
*Eugenia caryophyllus*	15.6 ± 0.2^c^	14.7 ± 0.1^d^	12.5 ± 0.2^d^	7.8 ± 0.1^d^	–	9.9 ± 0.3^e^	9.3 ± 0.2^d^	8.5 ± 0.1^d^	7.0 ± 0.1^c^	–
*Kaempferia galanga*	–	–	–	–	–	–	–	–	–	–
*Origanum compactum*	27.3 ± 0.5^f^	25.3 ± 0.4^e^	20.8 ± 0.7^e^	14.2 ± 0.9^e^	8.4 ± 0.2^b^	9.5 ± 0.4^b^	9.2 ± 0.0^d^	8.3 ± 0.2^df^	7.4 ± 0.2^d^	7.1 ± 0.2^b^
*Origanum heracleoticum*	30.3 ± 0.8 ^g^	29.0 ± 0.4^f^	25.8 ± 0.2^f^	22.3 ± 0.8^f^	11.7 ± 0.9^c^	12.3 ± 0.5^f^	11.8 ± 0.3^e^	10.5 ± 0.2^e^	9.4 ± 0.1^e^	8.7 ± 0.1^a^
*Origanum majorana*	16.7 ± 0.2^d^	12.0 ± 0.2^g^	–	–	–	15.9 ± 0.6^g^	12.9 ± 0.2^f^	8.1 ± 0.4^f^	7.7 ± 0.2^d^	–
*Salvia officinalis*	14.0 ± 0.2^h^	12.0 ± 0.4^g^	7.2 ± 0.1^g^	–	–	–	–	–	–	–
*Salvia sclarea*	10.5 ± 0.2^i^	9.4 ± 0.2^h^	–	–	–	–	–	–	–	–
*Thymus capitatus*	23.5 ± 0.6^j^	21.5 ± 0.5^i^	18.7 ± 0.4^h^	16.4 ± 0.2^g^	12.8 ± 0.1^d^	11.7 ± 0.3^h^	11.0 ± 0.1^g^	9.5 ± 0.1^g^	9.2 ± 0.2^e^	8.3 ± 0.1^c^
*Thymus mastichina*	11.9 ± 0.4^k^	10.4 ± 0.3^j^	7.1 ± 0.1^g^	–	–	–	–	–	–	–
*Thymus vulgaris thymoliferum*	29.6 ± 0.8 ^l^	27.3 ± 0.5^k^	24.7 ± 0.4^i^	18.2 ± 1.2^h^	8.9 ± 0.2^b^	11.0 ± 0.3^i^	10.2 ± 0.2^c^	8.4 ± 0.0^df^	7.6 ± 0.2^d^	7.1 ± 0.3^b^
Ampicillin^3^	31.0 ± 1.1	ND	ND	ND	ND	–	ND	ND	ND	ND
Chloramphenicol^3^	29.8 ± 0.4	ND	ND	ND	ND	–	ND	ND	ND	ND
Streptomycin^3^	–	ND	ND	ND	ND	12.4 ± 0.4	ND	ND	ND	ND
Blank control (ethanol)	–	–	–	–	–	–	–	–	–	–

^(–)^Diameter of inhibitory zone <7 mm considered as no antimicrobial activity. ND, not determined.

1 Concentrations (1, 1/2, 1/10, 1/20, 1/40) used were v/v.

2 Values are mean diameter of inhibitory zone (mm) ±SD of five replicates, followed by different letters in column are significantly different (*P* < 0.05). The diameter of paper disk (6 mm) is included.

3 Ampicillin (10 *μ*g), chloramphenicol (30 *μ*g), and streptomycin (10 *μ*g) used as positive control.

### Antimicrobial activity of essential oils components

**Figure 1:**
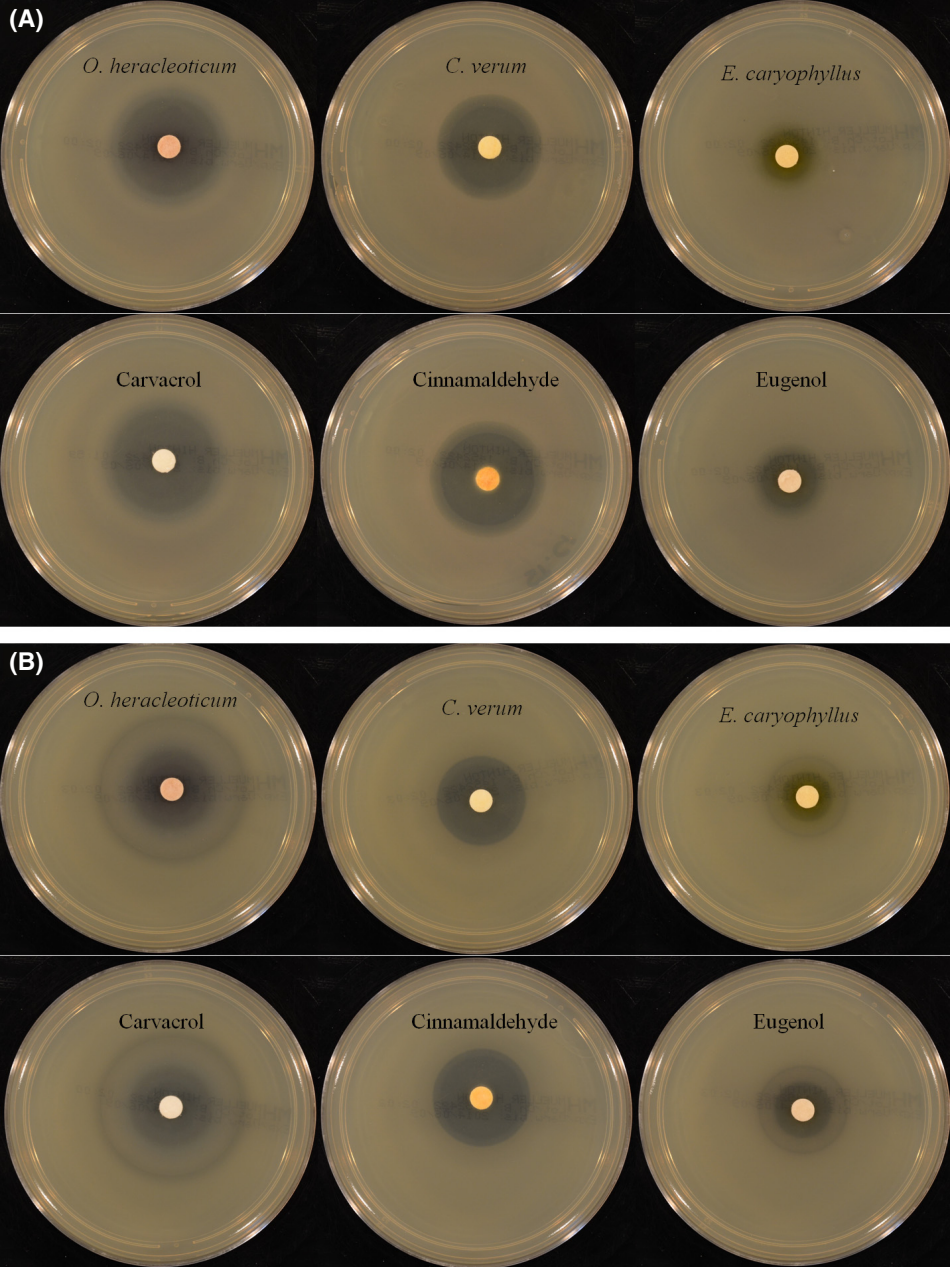
Inhibition diameter zones obtained by paper disk diffusion method for *Origanum heracleoticum*, *Cinnamomum verum*, *Eugenia caryophyllus*, carvacrol, cinnamaldehyde, and eugenol against (A) *Salmonella* Typhimurium ATCC 14028 and (B) *Pseudomonas fluorescens* ATCC 13525.

Some standard components such as carvacrol, cinnamaldehyde, eugenol, linalool, and thymol were tested under identical conditions (Table 6). As the main constituents in some essential oils, these components have been proven to be particularly effective against some species of Gram-positive and Gram-negative bacteria (Cosentino et al. 1999; Dorman and Deans 2000; Bagamboula et al. 2004; Kotan et al. 2007; Shan et al. 2007; Hussain et al. 2008; Castilho et al. 2012). The oxygenated components, *trans*-cinnamaldehyde, carvacrol, and thymol were shown in this study to possess stronger antibacterial activity in comparison with eugenol and linalool, which could explain the high antimicrobial activity of cinnamon, oregano, and thyme oils (Aligiannis et al. 2001; Baydar et al. 2004; Shan et al. 2007; Castilho et al. 2012). Cinnamaldehyde exhibited high levels of antimicrobial activity against all tested strains, whereas carvacrol and thymol, with the only exception against *P. flurorescens*, showed a lower activity. Figure 1 shows typical inhibition halos obtained for *O. heracleoticum*, *C. verum*, *E. caryophyllus*, carvacrol, cinnamaldehyde, and eugenol against *S*. Typhimurium and *P. fluorescens*.

**Table 6:** Inhibitory diameters and minimum inhibitory and bactericidal concentrations of essential oil constituents against food-borne and food spoilage bacteria

	Tested bacteria							
	
Samples	*L. monocytogenes* NCTC 11994	*L. monocytogenes* S0580	*S*. Typhimurium ATCC 14028	*S*. Typhimurium S0584	*E. coli* O157:H7 ATCC 35150	*E. coli* O157:H7 S0575	*B. thermosphacta* ATCC 11509	*P. fluorescens* ATCC 13525
Paper disk diffusion method (inhibition diameter, mm)^1^								
*trans*-Cinnamaldehyde	31.7 ± 0.5^a^	35.5 ± 0.3^a^	28.9 ± 0.5^a^	30.9 ± 0.1^a^	29.6 ± 0.5^a^	30.1 ± 0.2^a^	30.7 ± 0.5^a^	28.9 ± 0.7^a^
Carvacrol	27.3 ± 0.3^b^	30.0 ± 0.8^b^	21.7 ± 0.2^b^	30.4 ± 0.9^a^	22.4 ± 0.3^b^	23.9 ± 0.4^b^	32.9 ± 0.4^b^	15.1 ± 0.7^b^
Eugenol	14.4 ± 0.2^c^	15.2 ± 0.3^c^	15.6 ± 0.2^b^	18.2 ± 0.7^b^	15.9 ± 0.4^c^	16.1 ± 0.4^c^	18.8 ± 0.3^c^	14.8 ± 0.4^b^
Linalool	11.4 ± 0.1^d^	12.8 ± 0.3^d^	10.5 ± 0.2^d^	16.1 ± 0.3^c^	12.4 ± 0.2^d^	13.7 ± 0.5^d^	15.5 ± 0.2^d^	8.7 ± 0.3^c^
Thymol	31.0 ± 0.2^a^	35.6 ± 0.4^a^	23.4 ± 0.5^e^	33.3 ± 0.6^d^	23.0 ± 0.4^b^	25.5 ± 0.6^e^	39.7 ± 0.4^e^	19.0 ± 0.5^d^
Minimum inhibitory and bacterial concentrations (MIC/MBC, *μ*L/mL)^2^								
*trans*-Cinnamaldehyde	0.125/0.5	0.25/0.5	0.25/0.25	0.125/0.25	0.125/0.25	0.125/0.25	0.125/1	0.25/0.5
Carvacrol	0.125/0.25	0.125/0.25	0.125/0.375	0.188/0.25	0.125/0.25	0.25/0.375	0.5/1	1/>1.5
Eugenol	0.5/1	0.5/1	1/1	0.5/1	0.5/>1.5	0.5/>1.5	1/>1.5	1/>1.5
Linalool	1/>1.5	0.75/>1.5	>1.5/>1.5	1/>1.5	1/1.5	1/1	1/>1.5	>1.5/>1.5
Thymol	0.25/0.5	0.25/0.5	0.25/0.5	0.313/0.375	0.25/0.25	0.25/0.313	0.5/1	1/>1.5

1 Values are mean diameter of inhibitory zone (mm) ±standard deviation of five replicates of each component, followed by different letters in a column are significantly different (*P* < 0.05). The diameter of paper disk (mm) is included.

2 Values are results of four replicates.

### Determination of MIC and MBC

The results reported above revealed the potential of some essential oils such as cinnamon, clove, oregano, and thyme as natural preservatives to control food pathogenic and spoilage bacteria. To achieve precisely the antimicrobial properties of essential oils for potential application in food preservation, determination of MICs and MBCs were necessarily performed on seven selected essential oils and five standard components. The results showed variable effects of essential oils and their components on the tested bacterial strains (Tables 6, 7). Oils of *C. cassia*, *C. verum,* and *T. vulgaris thymoliferum* showed again strong antimicrobial activities in inhibiting the growth of pathogenic and spoilage bacteria at MICs ≤ 1 *μ*L/mL. The bacterial growth was also inhibited by oils of *O. compactum* at MICs ≤ 0.5 *μ*L/mL and *E. caryophyllus* at MICs ≤ 1 *μ*L/mL except for *P. fluorescens*. The essential oils of *C. cassia*, *C. verum*, *O. compactum*, *O. heracleoticum*, *T. capitatus,* and *T. vulgaris thymoliferum* showed bactericidal effects at concentrations ≤1.5 *μ*L/mL. Among tested microorganisms, as previously observed with the paper disk diffusion method, *P. fluorescens* was the least sensitive as higher concentrations of essential oils were needed with MICs and MBCs ranging from 1 to 1.5 *μ*L/mL. By comparison to previously published studies, our findings presented discrepancy of antimicrobial activity of selected essential oils against food-borne and spoilage bacteria. It may be explained by the different composition and percentage content of active constituents in essential oils, which have been found to have an important role in slowing down or stopping the bacterial growth or killing the bacteria (Ouattara et al. 1997; Bozin et al. 2006). Some factors influencing this variation in composition can be species, subspecies or variety of plants (Sarac and Ugur 2008), geographical locations (Sarac and Ugur 2008; Mechergui et al. 2010), harvesting seasons (Hussain et al. 2008), drying methods (Di Cesare et al. 2003), and also extraction methods (Burt 2004; Karakaya et al. 2011). Moreover, the methods used to assess the antimicrobial activity could also affect the generated outputs (Hammer et al. 1999; Burt and Reinders 2003; Burt 2004). Other factors such as the choice of bacterial strains and their sensitivity, volume of inoculum, incubation time, and temperature should also be related to the variation in the experimental results (Smith-Palmer et al. 1998; Burt 2004; Bozin et al. 2006).

**Table 7:** Minimum inhibitory (MIC) and minimum bactericidal (MBC) concentrations of selected essential oils against food-borne and food spoilage bacteria

	Test bacteria (MIC/MBC (*μ*L/mL))^1^							
	
Essential oils	*L. monocytogenes* NCTC 11994	*L. monocytogenes* S0580	*S*. Typhimurium ATCC 14028	*S*. Typhimurium S0584	*E. coli* O157:H7 ATCC 35150	*E. coli* O157:H7 S0575	*B. thermosphacta* ATCC 11509	*P. fluorescens* ATCC 13525
*Cinnamomum cassia*	0.5/0.5	0.25/0.25	0.25/1	0.25/1	0.5/1	0.25/0.25	0.5/0.5	1/1
*Cinnamomum verum*	0.5/0.5	0.25/0.5	0.5/0.5	0.5/1	0.5/0.5	0.25/0.5	0.5/1	1/1.5
*Eugenia caryophyllus*	1/>1.5	1/>1.5	1/1.5	1/1.5	1/1	1/1	0.5/0.5	1.5/1.5
*Origanum compactum*	0.5/0.5	0.25/0.25	0.5/0.5	0.25/0.5	0.25/0.5	0.5/0.5	0.5/0.5	1.5/1.5
*Origanum heracleoticum*	0.25/0.25	0.25/0.25	0.125/0.125	0.25/0.25	0.25/0.25	0.25/0.25	0.5/0.5	1/1
*Thymus capitatus*	0.5/0.5	0.5/1	1/1	0.5/1.5	0.5/1	0.25/0.25	0.13/0.25	1/1
*Thymus vulgaris thymoliferum*	0.5/0.5	0.25/0.25	0.25/0.5	0.25/0.5	0.25/0.25	0.25/0.5	0.25/0.5	1.5/1.5

1 Values are results of four replicates.

Against the pathogenic *L. monocytogenes*, *S*. Typhimurium and *E. coli* O157:H7, the essential oil of oregano (*O. heracleoticum* and *O. compactum*), thyme (*T. vulgaris thymoliferum*), and cinnamon (*C. cassia* and *C. verum*) were all strongly active. Our findings indicated comparable or even better results by comparison to the outputs of Oussalah et al. (2007). As evidence, essential oils of *O. heracleoticum*, *O. compactum*, *T. vulgaris thymoliferum*, and *E. caryophyllus* showed their effectiveness against *L. monocytogenes* with MICs of at least two times lower that of Oussalah et al. (2007). This could be due to the higher content of main and active component in the essential oils, for instant, higher carvacrol 68% in *O. heracleoticum* oil to 54% in previous study of Oussalah et al. (2007), which could result in a better antilisterial activity. The results obtained with *L. monocytogenes* are very helpful and relevant as this microorganism can grow at refrigeration temperature, over a wide range of pH values above 4.4 and in the presence of high salt content surviving mild preservation treatment (Hazzit et al. 2006), features that make it difficult to eliminate this microorganism from foods. However, the results above were generated from two different methods, even inoculum concentrations. Therefore, bacterial sensibility to essential oil could be different (Hammer et al. 1999; Burt 2004). Consequently, these findings could be considered as extra confirmatory information in this study. In this study, antimicrobial effect against *S*. Typhimurium was ∼2- to 10-fold for oregano oil, 20-fold for clove oil and even 80-fold for thyme oil by comparison to findings of Hammer et al. (1999). Cinnamon oil also inactivated effectively the growth of pathogenic *S*. Typhimurium as similarly reported by Unlu et al. (2010). The oregano oils were much more effective than clove oil against *E. coli* O157:H7, which is similar to the findings of Oussalah et al. (2007), but completely opposed to the result of Moreira et al. (2005). This could be explained by the use of different bacterial strains of *E. coli* O157:H7, different methods for MIC and MBC determination and also different subspecies of oregano. Other authors also revealed the antimicrobial effects of these essential oils against different strains of *L. monocytogenes* (Lis-Balchin and Deans 1997; Faleiro et al. 2005), *Salmonella* (Özkan et al. 2003; Rota et al. 2008) and *E. coli* O157:H7 (Sağdıç et al. 2002; Özkan et al. 2003; Rota et al. 2008; Karakaya et al. 2011). Overall, the selective essential oils and their components exhibited a wide range of efficacy in inhibiting the pathogenic bacterial growths. Association of *L. monocytogenes*, *S*. Typhimurium, and enterohemorrhagic *E. coli* O157:H7 with food-borne outbreaks is well documented (Newell et al. 2010; EFSA and ECDC 2011). According to the broad spectrum against these food-borne pathogens, the use of these effective natural alternatives into foods could help food producers to shift away from artificial preservative and to reduce or even eliminate these food poisoning bacteria and control their contaminations in foods.

*Brochothrix thermosphacta* and *P. fluorescens* are commonly responsible for food spoilage causing off-odors, off-flavors, and slime production, especially in highly perishable products like meats and meat products. In this study, most of selected essential oils exhibited a remarkable activity against *B. thermosphacta*. These oils could potentially be good candidates in inhibiting the growth of *B. thermosphacta*. Apparently, Spanish oregano oil (*T. capitatus*) and thyme oil showed slightly better activity among tested oils. Only a few studies have been reported on the antimicrobial activity of such essential oils against *B. thermosphacta* (Ouattara et al. 1997; Baratta et al. 1998; Dorman and Deans 2000). Therefore, this study brings some interestingly complementary findings to the previously published work. Dorman and Deans (2000) have found qualitatively similar result to our finding. In contrast, Ouattara et al. (1997) demonstrated that cinnamon and clove oils were the most active, while oregano and thyme oil failed to inhibit bacterial growth. This discrepancy could be explained by a relationship between the inhibitory effect of essential oils and the presence of their active volatile constituents and sensitivity of different bacterial strains. On the other hand, Gram-negative *P. fluorescens* was observed as the least sensitive to majority of essential oils among the tested bacterial strains. This is in agreement with many studies having studied different strains of *Pseudomonas* other than *P. fluorescens* such as *Pseudomonas putida* (Oussalah et al. 2006) and *P. aeruginosa* (Ouattara et al. 1997; Cosentino et al. 1999; Hammer et al. 1999; Dorman and Deans 2000; Özkan et al. 2003; Prabuseenivasan et al. 2006; Bouhdid et al. 2008; Sarac and Ugur 2008; Unlu et al. 2010; Castilho et al. 2012). Only a few studies reported antimicrobial activities of essential oils, especially oregano and thyme oils from different species, against *P. fluorescens* and the resistance of this food spoilage bacterium is well-known (Baratta et al. 1998; Özkan et al. 2003; Sarac and Ugur 2008; Ruiz-Navajas et al. 2012). Our results proved that low antibacterial activity of carvacrol and thymol against *P. fluorescens* can explain the low activity of oregano and thyme oils comparing to other bacteria. The lower sensibility of this bacterium has been attributed to an active efflux mechanism and the barrier function of the outer membrane lipopolysaccharide, which can screen out and restricts entry of some antimicrobial agents or compounds (Cox and Markham 2007). However, essential oils from *C. cassia*, *C. verum* and their main constituent cinnamaldehyde clearly worked well against this food spoilage bacterium (Ouattara et al. 1997; Oussalah et al. 2006; Di Pasqua et al. 2007; Unlu et al. 2010). Thus, these substances could be potentially important to be used as antimicrobial agent in food preservation.

The essential oils and standard components were demonstrated to inhibit the growth of both food pathogenic and food spoilage bacteria. Thus, the data obtained in this study can be evidently served as a well confirmatory and complementary data to the previously published works.

## Conclusion

The commercial essential oils from cinnamon, oregano, and thyme exhibit promising antimicrobial effects against selected food-borne and food spoilage bacteria, which can be attributed to the presence of the principle bioactive constituents, especially cinnamaldehyde, carvacrol, and thymol. These investigated essential oils and their main active components could be potential candidates to be used as natural alternatives for further application in food preservation to retard or inhibit the bacterial growth and for safety and to extend the shelf life of the food products. However, the confirmation of antimicrobial efficiency and organoleptic impact of these essential oils in foodstuffs need to be evaluated.
